# Morphological characteristics of spiral tibial shaft fractures involving the distal articular surface: a retrospective observational study

**DOI:** 10.1038/s41598-025-19668-2

**Published:** 2025-10-10

**Authors:** Xianjie Ai, Yu Su, Yujie Li, Hongfei Qi, Taotao Ren, Zhimeng Wang, Zhong Li, Bo Wu, Ming Li

**Affiliations:** 1 Lower Extremity Division, Orthopedic Trauma Department, Honghui Hospital, Xi’an Jiaotong University, Youyi East Road No.555, Beilin District, Xi’an, China; 2 Medical College of Yan’an University, No. 580, Shengdi Road, Bao Ta District, Yan’an, China; 3 Yulin Hospital, The First Affiliated Hospital of Xi’an Jiaotong University, Yulin, China

**Keywords:** Spiral tibial shaft fracture, Distal articular surface, Fracture line distribution, Heat map analysis, Anatomy, Medical research, Image processing

## Abstract

Spiral fractures of the tibial shaft are frequently accompanied by injuries involving the distal articular surface; however, comprehensive investigations into the morphological characteristics of fracture lines extending to the joint surface remain limited. Existing classification systems are insufficient to comprehensively characterize the continuum of injuries spanning from the diaphysis to the articular surface. This study aimed to delineate the extension patterns of spiral tibial shaft fractures toward the distal articular surface, quantify the frequency of involvement across distinct anatomical regions, and characterize their spatial distribution, thereby providing a morphological basis for more precise diagnosis and treatment. A single-center retrospective cohort comprising 160 patients with spiral tibial shaft fractures treated at the Xi’an Jiaotong University Affiliated Honghui Hospital between May 2020 and December 2024 was included. Computed tomography (CT) images were independently screened and assessed by three senior physicians blinded to clinical data, with AO/OTA classification demonstrating excellent inter-rater reliability (κ > 0.80). Among these, 117 cases exhibited involvement of the distal articular surface. Fracture lines were registered onto a standardized tibial template utilizing a processing pipeline integrating Mimics, 3-Matic, NX, AutoCAD, and Origin software, enabling the generation of three-dimensional fracture line distribution maps and heatmaps. Among the 160 cases of spiral tibial shaft fractures, 117 (73.1%) exhibited fracture lines involving the distal articular surface. The specific distribution was as follows: 85 cases (72.6%) involved the posterior malleolus, 46 cases (39.3%) the anterior malleolus, and 29 cases (24.9%) the medial malleolus. Distribution maps revealed that 57 cases (48.7%) involved only the posterior malleolus, 16 cases (13.7%) only the anterior malleolus, and 9 cases (7.7%) only the medial malleolus; 15 cases (12.8%) involved both the posterior and anterior malleoli, 5 cases (4.3%) both the posterior and medial malleoli, and 7 cases (6.0%) both the anterior and medial malleoli. In comparison, 8 cases (6.8%) exhibited involvement of all three regions. Heatmap analysis revealed a highly modular distribution of fracture lines across the distal articular surface. The highest density was observed in the posterior malleolar region, forming an arc-shaped high-density zone. In the anterior malleolar region, two linear high-density bands were primarily located along the margins of the anterior malleolar module and the Chaput tubercle. Fracture lines in the medial malleolus were predominantly concentrated at the junction between the medial malleolar prominence and the articular surface. Spiral tibial shaft fractures frequently extend into the distal articular surface, exhibiting diverse injury patterns that go beyond the traditionally recognized posterior malleolar involvement. Notably, anterior malleolar fractures have been consistently underestimated. Existing classification systems fail to adequately capture the continuity of injuries spanning from the diaphysis to the articular surface. The fracture line distribution maps and heatmaps presented in this study illustrate a modular, region-specific pattern across the distal articular surface. These visualizations provide a foundational framework for developing an integrated classification system encompassing both the tibial shaft and ankle joint, thereby informing more refined surgical strategies and improving treatment outcomes and safety.

## Introduction

Spiral tibial shaft fractures represent a distinct fracture pattern caused by torsional forces. Previous studies have demonstrated a close association between such fractures and concomitant ankle joint injuries^1^particularly when the distal third of the tibial diaphysis is affected by a spiral or oblique fracture^2–4^. These injuries may manifest either as an extension of the primary fracture line into the articular surface or as separate, non-contiguous fractures unrelated to the initial diaphyseal injury^4,5^. Consequently, accurate assessment of articular surface involvement is essential for guiding appropriate treatment decisions.

The sensitivity of imaging modalities plays a pivotal role in the detection of associated articular injuries. For instance, Chen et al. reported that among 28 cases of distal third spiral tibial fractures in a cohort of 128 patients, posterior malleolar fractures were identified by radiographs in only 10 cases^2^. Similarly, Warner et al. found that plain radiographs detected posterior malleolar involvement in merely 28% (7/24) of cases. In contrast, the detection rate increased to 56% (14/25) with computed tomography (CT), and further improved to 84% (21/25) when combined with magnetic resonance imaging (MRI)^3^. Additional studies have shown that the misdiagnosis rate of posterior malleolar fractures in spiral tibial shaft fractures by plain radiographs may range from 67.9 to 91.2%, underscoring the inadequacy of radiography alone for reliable diagnosis^6^. The incorporation of CT imaging significantly enhances diagnostic accuracy for these occult injuries^2,7,8^. A prospective study by Lisitano et al. further demonstrated that plain radiographs are insufficient for reliably detecting non-displaced posterior malleolar fractures associated with spiral tibial shaft fractures^9^. Consequently, CT imaging has increasingly been adopted as a standard adjunct in the evaluation of spiral tibial shaft fractures.

Despite the widespread application of high-resolution CT imaging, the current understanding of articular involvement in spiral tibial shaft fractures remains predominantly centered on the posterior malleolus. For example, Kin et al.^10^ reported ankle joint involvement in 64.7% of spiral tibial shaft fractures, while Phillip et al.^11^ found that 68.9% of 84 distal tibial fractures involved the articular surface, with posterior malleolar fractures accounting for 48.4% of cases. A retrospective study by Wang et al.^12^ further demonstrated that posterior malleolar involvement was present in up to 74% of isolated distal tibial fractures. Although recent morphological analyses have suggested that fracture lines may also extend to the anterior and medial malleoli^13^ systematic data regarding the incidence, spatial distribution, and clinical relevance of these regions remain scarce. This knowledge gap hinders a comprehensive understanding of articular surface involvement in spiral tibial shaft fractures. Moreover, existing ankle fracture classification systems primarily focus on the posterior malleolus or the ankle joint itself, making them insufficient for capturing complex injury patterns characterized by primary diaphyseal fractures with fracture lines extending distally into the joint surface or accompanied by occult intra-articular fissures. There is an urgent need to establish an integrated classification framework that encompasses the full spectrum of diaphyseal-to-articular injuries.

This knowledge gap has directly impeded the precision of clinical diagnosis and treatment planning. The present study aims to systematically characterize the three-dimensional extension patterns of spiral tibial shaft fractures into the distal articular surface. By quantitatively analyzing the frequency and distribution of fracture lines involving the posterior, anterior, and medial malleoli, this study seeks to address the blind spots inherent in the prevailing posterior–malleolus–centric perspective. The findings are expected to provide both imaging-based and theoretical foundations for accurately delineating high-risk regions and formulating individualized therapeutic strategies. Moreover, the morphological insights gained may serve as the basis for establishing a novel, integrated classification framework encompassing injuries of both the tibial shaft and the articular surface.

## Method

This single-center retrospective observational study was approved by the Ethics Committee of Xi’an Honghui Hospital (Approval No. 202410008) and conducted by the STROBE guidelines for observational studies^14^. All experiments involving human participants were performed with the informed consent of the patients. All research methods strictly adhered to the ethical requirements of the Declaration of Helsinki and complied with relevant ethical standards throughout the study. The study cohort comprised CT imaging data from 160 patients diagnosed with primarily tibial shaft spiral fractures at the Department of Trauma and Orthopedics of our institution between May 2020 and December 2024.

### Research qualification standards

Inclusion criteria:

1.Age ≥ 18 years; 2.Patients with closed tibial shaft fractures; 3. Primarily spiral tibial shaft fractures classified as AO/OTA types 42A1, 42B1, 42C1, or 43A1, with fracture lines extending to the distal articular surface or exhibiting occult intra-articular extensions; 4. Preoperative high-quality CT imaging and CT data are suitable for three-dimensional reconstruction.

Exclusion criteria:

1. Pathological fractures; 2.Open fractures; 3. Concomitant Pilon fractures (AO/OTA types 43B or 43 C) were excluded; 4. History of ipsilateral ankle joint deformity.

### Data collection

Demographic and clinical information, including sex, age, height, weight, and mechanism of injury, was extracted from the electronic medical records. DICOM (Digital Imaging and Communications in Medicine) data for computed tomography (CT) were retrieved from the hospital’s PACS (Picture Archiving and Communication System). CT scans were performed with the following parameters: tube voltage of 120 kV, tube current of 150 mA, and slice thickness of 1 mm. The scan range encompassed the entire length of the tibia.

### Fracture classification and interobserver agreement

Three observers (Z.W., B.W., and M.L.), each with over five years of experience in orthopedic trauma, independently evaluated all preoperative CT scans under blinded conditions. Prior to the formal assessment, classification boundaries were standardized using 20 non-study sample cases, which were re-evaluated after a 4-week interval to minimize recall bias. Pairwise interobserver agreement was calculated using Cohen’s κ, while overall agreement among all three observers was assessed using Fleiss’ κ_F. The interpretation of κ values followed the criteria proposed by Landis and Koch.

### Generation of fracture line distribution maps and heatmaps

**Fig. 1:**
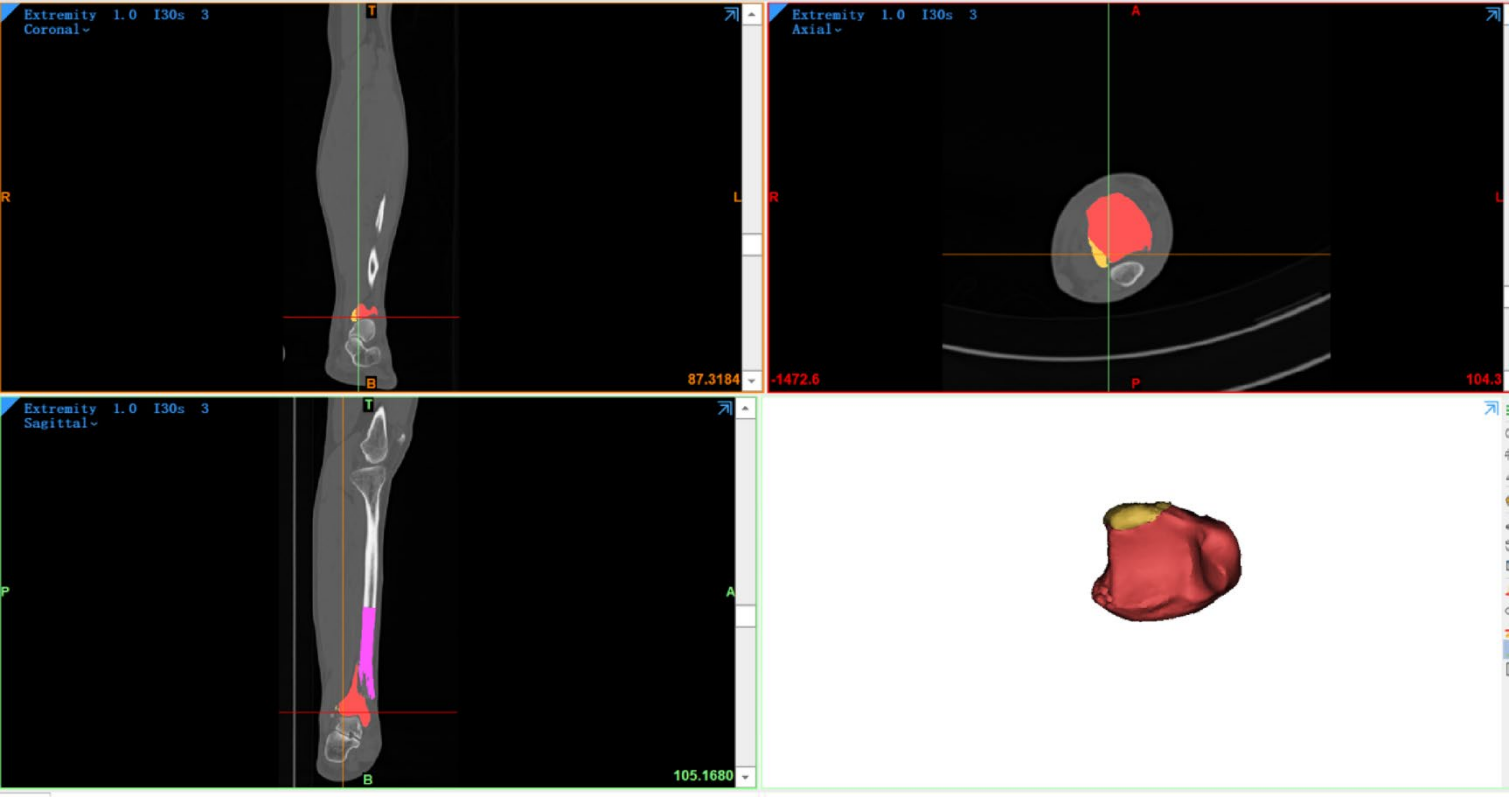
Three-dimensional fracture reconstruction.

**Fig. 2:**
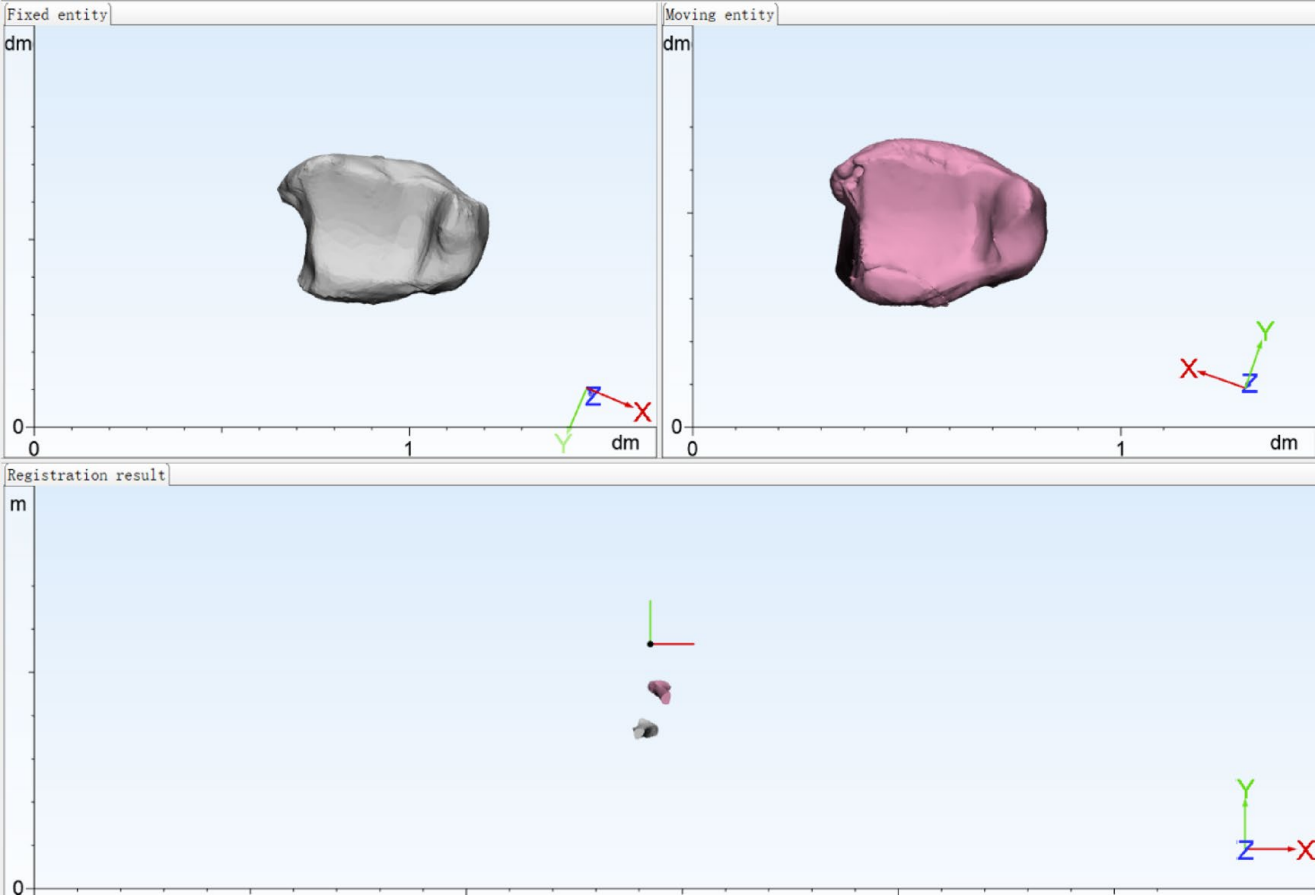
Registration.

**Fig. 3:**
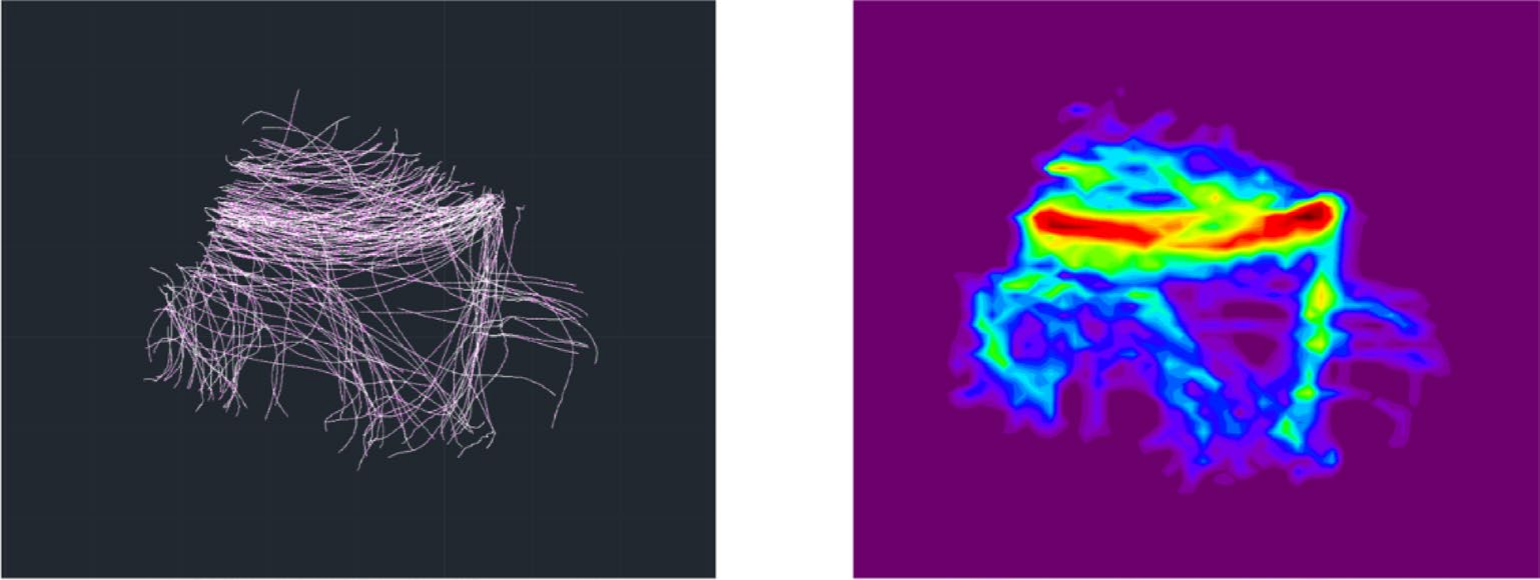
Fracture line extraction and heat map generation.

We adopted a previously validated technique for fracture line mapping and heatmap construction^15^. The workflow was as follows:

1. A standardized template of the middle-to-distal third of a healthy adult tibia was reconstructed in three dimensions using Mimics 21.0 (Materialise, Leuven, Belgium).
2. CT data from 117 spiral tibial fractures involving the ankle joint were imported into Mimics, and virtual fracture reduction was performed for each case (Fig. 1).

3. In 3-Matic 13.0 (Materialise, Leuven, Belgium), each reconstructed fracture model was rigidly registered to the tibial template using five standardized views: anterior, posterior, medial, lateral, and distal (Fig. 2).

4. Three-dimensional fracture lines were precisely traced onto the template using Unigraphics NX 12.0 (Siemens PLM Software Inc., Plano, TX, USA) and exported as IGES (Initial Graphics Exchange Specification) files.
5. The IGES-format fracture lines were imported into 3-Matic and superimposed onto the template to generate an integrated 3D fracture line distribution map.
6. Spatial coordinates of the fracture lines were extracted at 0.1 mm intervals using AutoCAD 2020 (Autodesk Inc., San Rafael, CA, USA). Kernel density estimation was then performed in Origin 2024 (OriginLab, Northampton, MA, USA) to construct heatmaps (Fig. 3), which were subsequently mapped back onto the template to depict areas of high and low fracture line density visually.

### Data analysis

Descriptive statistics were used in this study: continuous variables were presented as mean ± standard deviation (SD), while categorical variables were expressed as frequencies and proportions. Fracture line morphology was illustrated using distribution maps, and spatial heterogeneity was visualized via heatmaps. Interobserver agreement for AO/OTA classification was first assessed using pairwise Cohen’s κ coefficients, followed by overall agreement evaluation using Fleiss’ κ_F. The interpretation of κ values was based on the Landis and Koch criteria, with values between 0.81 and 1.00 indicating almost perfect agreement. The 95% confidence interval (CI) for κ_F was estimated using 1,000 bootstrap resampling iterations. All statistical analyses were performed using SPSS version 29.0 and R version 4.3.0 (irr package).

## Results

**Fig. 4:**
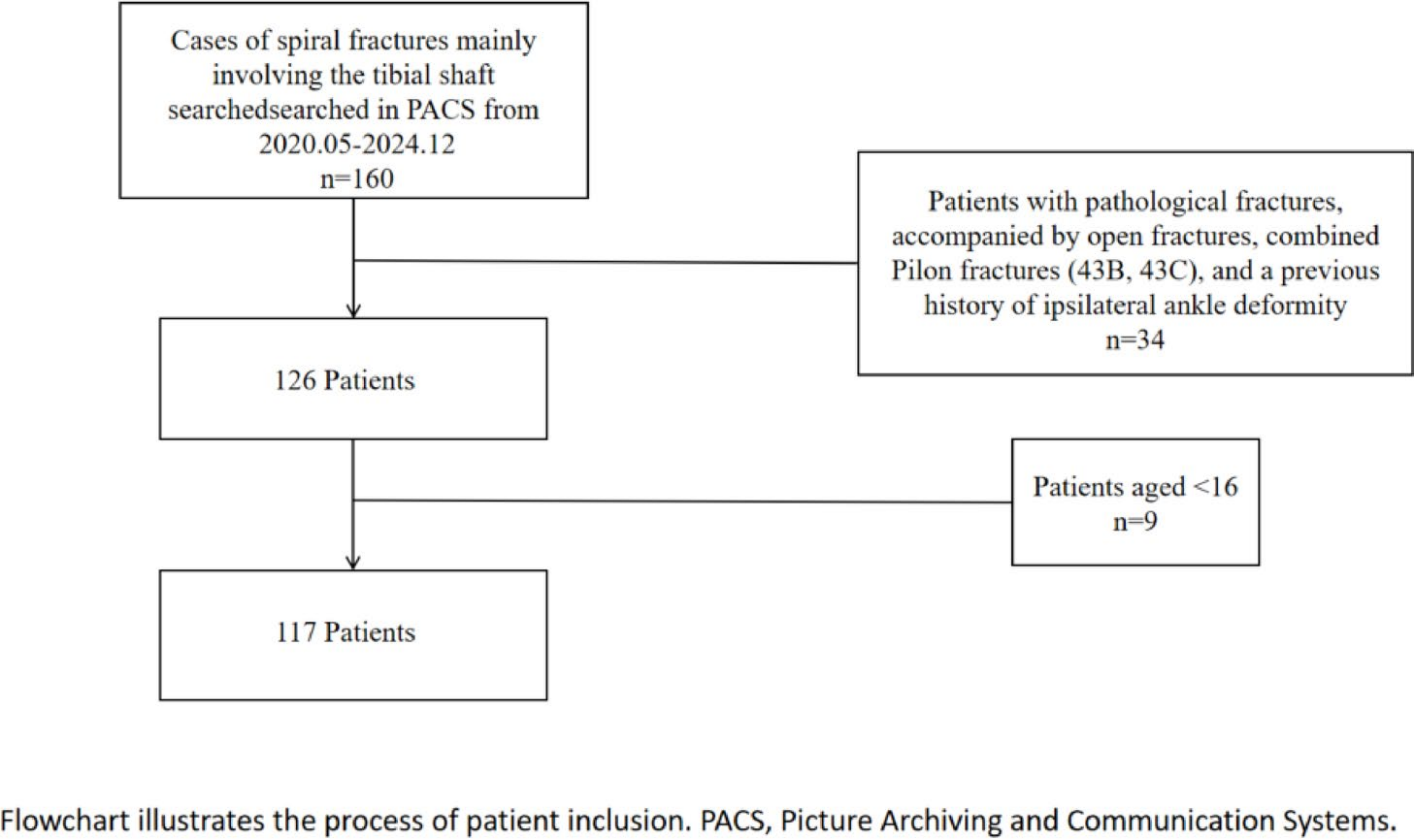
Flowchart illustrates the process of patient inclusion.

From May 2020 to December 2024, a total of 160 patients with spiral tibial fractures were identified through PACS. Patients with pathological fractures, open fractures, Pilon fractures, or a prior history of ipsilateral ankle deformity were excluded. Additionally, 34 patients were excluded based on exclusion criteria, including nine individuals under the age of 16 (Fig. 4).

A total of 117 cases (73.1%) were confirmed as primarily spiral tibial shaft fractures (AO/OTA types 42A1, 42B1, 42C1, and 43A1) with fracture lines involving the distal articular surface or occult intra-articular extension (Table 1). Among these 117 patients, 76 were male and 41 were female, with an age range of 18 to 80 years and a mean age of 46.93 ± 14.94 years (Table 2).

**Table 1:** AO/OTA classification and the involvement of the fracture area.

AO/OTA	Isolated Posterior Malleolar (*n*/%)	Isolated Anterior Malleolar(*n*/%)	Isolated Medial Malleolar(*n*/%)	Posterior + Anterior Malleolus(*n*/%)	Posterior + Medial Malleolus(*n*/%)	Anterior + Medial Malleolus(*n*/%)	Posterior + Anterior + Medial Malleolus(*n*/%)	Total(*n*/%)
42A1	36(30.8%)	8(6.8%)	2(1.7%)	8(6.8%)	2(1.7%)	0(0%)	3(2.6%)	59(50.4%)
42B1	12(10.3%)	3(2.6%)	4(3.4%)	5(4.3%)	1(0.9%)	4(3.4%)	3(2.6%)	32(27.4%)
42C1	5(4.3%)	0(0%)	1(0.9%)	1(0.9%)	1(0.9%)	2(1.7%)	0(0%)	10(8.5%)
43A1	4(3.4%)	5(4.3%)	2(1.7%)	1(0.9%)	1(0.9%)	1(0.9%)	2(1.7%)	16(13.7%)
Total	57(48.7%)	16(13.7%)	9(7.7%)	15(12.8%)	5(4.3%)	7(6.0%)	8(6.8%)	117(100%)

**Table 2:** Basic population information Sheet.

Character	Data (*n* = 117)	Type of Injury	Data (*n* = 117)
Male	76(65%)	Car accident	15
Female	41(35%)	Slip and fall	61
Age	46.93 ± 14.94 years (18–80)	Riding a motorbike	27
		Walking down stairs	7
		Heavy objects	4
		Falling	3

Interobserver agreement among the three independent evaluators for AO/OTA classification and determination of articular involvement exceeded a Kappa value of 0.80, indicating “almost perfect agreement” according to Landis and Koch criteria (Table 3). These results demonstrate the high reliability of imaging assessments in this study and validate their use for subsequent statistical analyses and clinical interpretation.

**Table 3:** Consistency test.

	Observers	κ/ICC	95% CI	Level of agreement*
AO/OTA classification	A vs. B	0.87	0.82–0.92	Almost perfect
	A vs. C	0.85	0.79–0.91	Almost perfect
	B vs. C	0.86	0.80–0.92	Almost perfect
	Fleiss’ κ (overall)	0.86	0.82–0.90	Almost perfect
Posterior malleolus involvement	Fleiss’ κ	0.91	0.87–0.95	Almost perfect
Anterior malleolus involvement	Fleiss’ κ	0.83	0.78–0.88	Almost perfect
Medial malleolus involvement	Fleiss’ κ	0.88	0.83–0.93	Almost perfect

*According to Landis & Koch criteria.

### Fracture line distribution and heatmap analysis

**Fig. 5:**
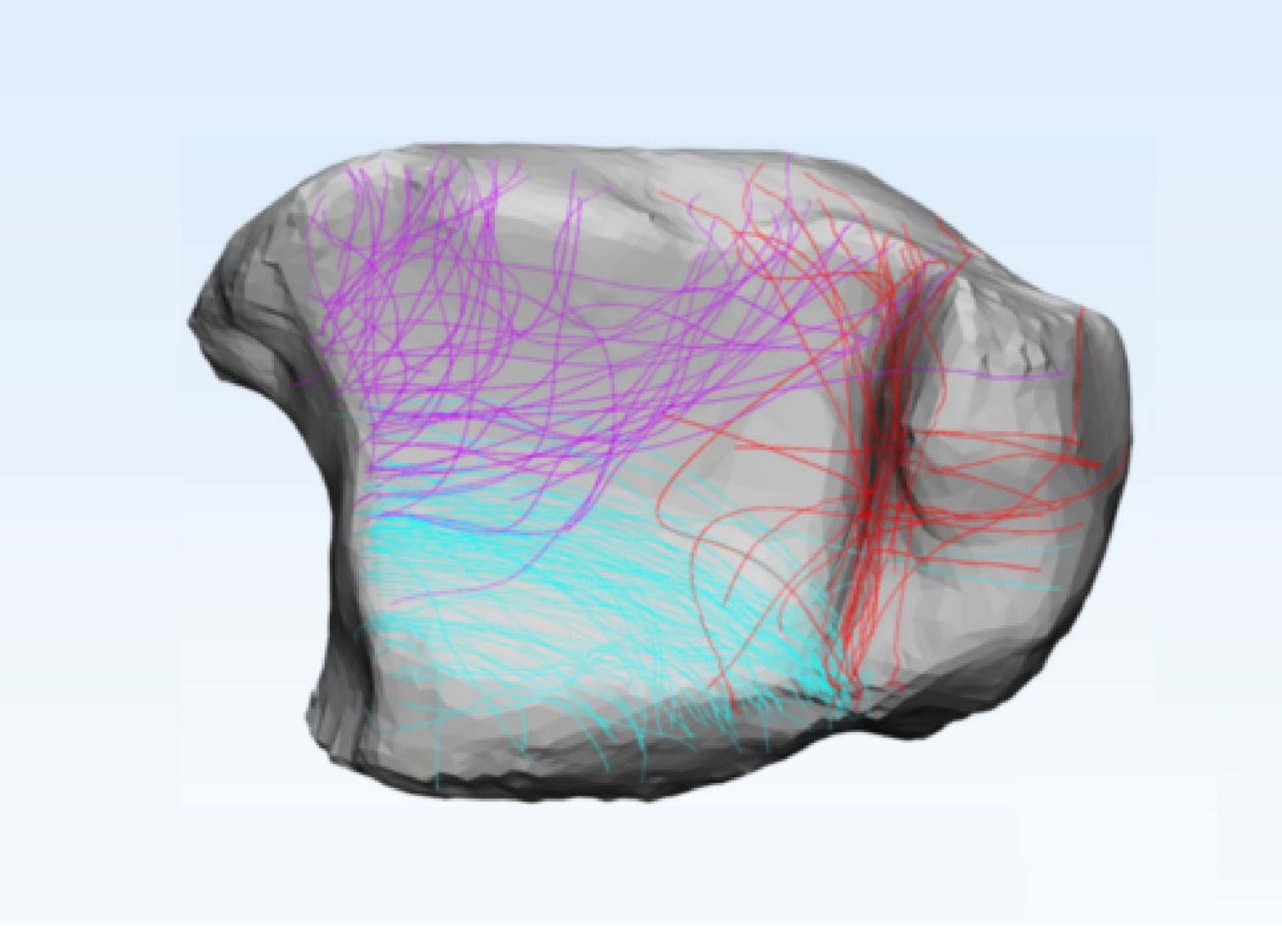
Fracture line distribution map.

**Fig. 6:**
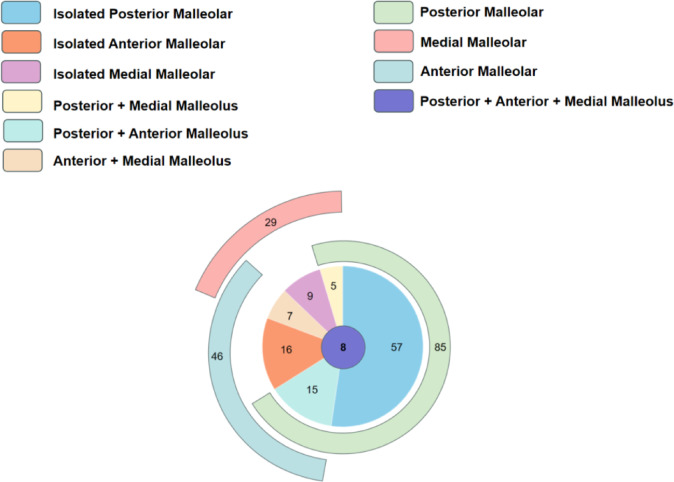
Fracture line involvement in different areas.

Fracture line distribution mapping revealed that among the 117 cases with articular involvement, fracture lines extended into the posterior malleolar region in 85 cases (72.6%), the anterior malleolar region in 46 cases (39.3%), and the medial malleolar region in 29 cases (24.9%) (Fig. 5). Based on the specific pattern of articular involvement, these 117 cases were further classified into seven subtypes (Table 4; Fig. 6):

**Table 4:** Frequency and incidence distribution of spiral tibial shaft fractures involving different regions of the distal Joint.

Region of involvement	Frequency	Incidence
Posterior Malleolar	85	72.6%
Anterior Malleolar	46	39.3%
Medial Malleolar	29	24.9%
Isolated Posterior Malleolar	57	48.7%
Isolated Anterior Malleolar	16	13.7%
Isolated Medial Malleolar	9	7.7%
Posterior + Anterior Malleolus	15	12.8%
Posterior + Medial Malleolus	5	4.3%
Anterior + Medial Malleolus	7	6.0%
Posterior + Anterior + Medial Malleolus	8	6.8%

1. Posterior malleolus only: 57 cases.
2. Anterior malleolus only: 16 cases.
3. Medial malleolus only: 9 cases.
4. Posterior and anterior malleoli: 15 cases.
5. Posterior and medial malleoli: 5 cases.
6. Anterior and medial malleoli: 7 cases.
7. Involvement of all three regions: 8 cases.

**Fig. 7:**
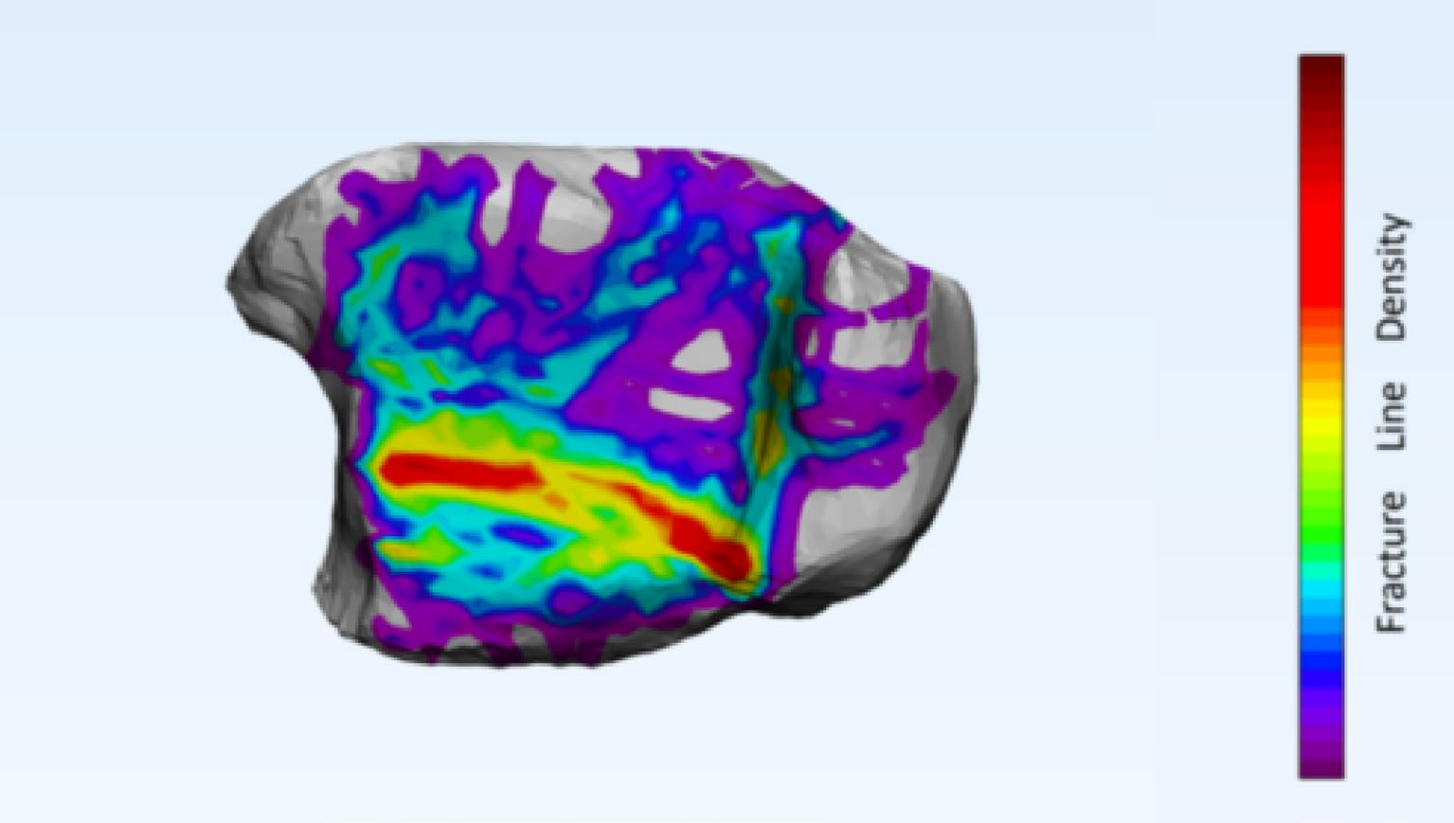
Heat map of fracture lines.

The fracture line heatmap demonstrated a highly modular and region-specific distribution across the distal articular surface. The posterior malleolar region exhibited the highest fracture line density, with widely distributed lines forming an almost arc-shaped high-density zone within the posterior malleolar module. In the anterior malleolar region, fracture lines were also relatively dense, predominantly located along the interface between the anterior malleolar and Chaput modules, forming two distinct linear high-density bands. In contrast, fracture lines in the medial malleolar region were more localized, primarily affecting the junction between the medial malleolar prominence and the articular surface (Fig. 7).

## Discussion

The fractures included in this study were defined as primarily diaphyseal spiral tibial fractures, corresponding to AO/OTA classifications 42A1, 42B1, 42C1, and 43A1^16^, which are commonly associated with an axial rotational injury mechanism^17^. Unlike typical pilon fractures (43B and 43 C) that result from vertical axial compression and often present with comminution or articular surface impaction, these spiral fractures frequently demonstrate extension of the fracture line toward the distal articular surface, thereby involving the ankle joint without vertical impaction characteristics^5,17,18^. With growing interest in the morphological characteristics of spiral tibial shaft fractures, a more straightforward clinical implication has emerged. When fracture lines extend into the distal tibial articular surface, simultaneous fixation of intra-articular fragments should be prioritized alongside intramedullary nailing for diaphyseal stabilization. This combined approach allows for restoration of joint congruity without significantly increasing surgical trauma and has been reported to reduce the risk of subsequent joint degeneration^19–21^. However, the success of this technique depends heavily on a comprehensive understanding of the spatial pattern and distribution of articular involvement in such fractures.

Numerous scholars have recommended incorporating computed tomography (CT) into the preoperative assessment of spiral tibial shaft fractures in addition to conventional radiography^7,9,10^. Consistent with this view, the present study also advocates CT evaluation to determine whether the fracture line involves the articular surface. While prior research has primarily focused on posterior malleolar involvement in such spiral tibial shaft fractures, relatively limited attention has been paid to the extension patterns toward other regions of the distal tibial articular surface.

More importantly, existing classification systems—such as the Weber, Lauge-Hansen, and AO/OTA systems for ankle fractures, as well as the Haraguchi, Mason, and Bartoníček/Rammelt systems for posterior malleolar fractures—were all developed with the ankle joint as their focal point. These systems lack a comprehensive framework to describe composite fractures in which a primary tibial shaft fracture line extends into or is accompanied by subtle, often occult, intra-articular involvement of the distal tibia.

In this study, a total of 160 cases of spiral tibial shaft fractures were retrospectively collected. All patients underwent both conventional radiography and preoperative CT. Fracture imaging and AO/OTA classification were independently assessed by three observers (Z.W., B.W., and M.L.) (Table 1). The results demonstrated that 117 of the 160 cases (73.1%) exhibited articular involvement, a rate consistent with previous reports^10,11,21^. Of particular note is that such spiral fractures affecting both the tibial shaft and ankle joint are coded separately for diaphyseal and distal segments under the current AO/OTA system. This segmented approach fails to capture the continuous nature of the injury. In our dataset, marked differences were observed in the distribution of articular involvement across AO/OTA subtypes. For instance, among 42A1-type fractures, 36 cases (30.8%) demonstrated isolated posterior malleolar involvement—substantially higher than in other subtypes—suggesting subtype-specific biomechanical transmission mechanisms. Accurate identification of these complex fractures is crucial not only for determining the fixation strategy but also for prognostic evaluation. Therefore, we argue that it is essential to move beyond the traditional “ankle-centered” classification perspective and to develop a unified system that integrates injuries of the tibial shaft and ankle joint into a cohesive framework.

To comprehensively characterize the involvement of the distal articular surface, this study employed a validated fracture mapping technique^22^ to visualize the fracture patterns in all 117 cases with intra-articular extension. Previous studies have suggested that spiral tibial shaft fractures frequently involve the posterior or medial malleolus^4,5^. Consistent with these findings, the present analysis revealed posterior malleolar involvement in 85 cases (72.6%) and medial malleolar extension in 29 cases (24.9%). Notably, anterior malleolar involvement was also identified in 46 cases (39.3%), a finding that has received limited attention in prior literature. To quantitatively characterize the distribution pattern of fracture lines, a density heatmap was superimposed on the fracture line distribution map^23^. The color gradient revealed an arc-shaped high-density band in the posterior malleolar region, with the broadest extent and highest density, closely corresponding to the pronation-external rotation ankle fracture pattern described by Yu et al.^24^. In the anterior malleolar region, two parallel high-density bands were observed along the borders of the anterior malleolar and Chaput modules. The medial malleolar region displayed a more localized distribution, with a density peak concentrated at the junction of the medial malleolar tip and the articular surface. The heatmap not only delineates high-risk zones of articular involvement in spiral tibial shaft fractures but also reinforces the clinical significance of anterior malleolar fractures. Despite the posterior malleolus being more frequently involved, anterior lesions warrant equal attention during preoperative assessment.

Based on the quantitative morphological analysis of the distal articular surface and the high-density heatmap of spiral tibial shaft fractures, this study is the first to systematically delineate the spatial trajectory of fracture lines extending from the diaphysis to the articular surface, precisely identifying high-risk zones at the posterior, anterior, and medial malleoli. This finding not only provides a panoramic depiction of the spatial continuity between diaphyseal and articular injuries but also establishes a precise and modular reference framework for developing an integrated classification system. Furthermore, the identification of high-risk zones with distinct modular patterns offers a practical imaging-based tool to support the preoperative planning of individualized and precision-oriented surgical strategies. Such an approach holds promise for reducing intraoperative uncertainty, minimizing complications, and improving overall clinical outcomes.

Moreover, this study identified a notably high incidence of anterior malleolar involvement in spiral tibial shaft fractures, posing new challenges for clinical diagnosis and treatment. In our cohort, 39.3% of patients exhibited anterior malleolar extension, a finding largely overlooked in previous literature. The anterior and posterior malleoli together constitute the distal tibial articular surface, and fracture lines traversing these regions directly compromise joint stability. Currently, closed reduction and intramedullary nailing (IMN) is widely accepted as the standard treatment for tibial shaft fractures, owing to its ability to provide stable fixation, promote fracture healing, and reduce complications^25^. However, IMN may inadvertently cause secondary injury to occult or unfixed distal articular fragments. Literature reports that posterior malleolar fractures associated with tibial shaft fractures often present occultly^26^leading to frequent underdiagnosis in clinical practice. Consequently, during IMN treatment, secondary displacement of posterior malleolar fragments may occur, resulting in severe adverse clinical outcomes^8,10,21^. This risk of displacement is not limited to the intraoperative period but may also manifest during patient rehabilitation and early weight-bearing stages. Although anterior malleolar involvement has received comparatively less attention, neglecting its presence can similarly lead to displacement, nonunion, malunion, and postoperative complications during IMN fixation. Key surgical steps such as reaming and nail insertion may exacerbate these risks if preoperative diagnosis is incomplete or intraoperative fixation of anterior malleolar fragments is inadequate. Therefore, we advocate that clinicians maintain heightened vigilance regarding the high prevalence of anterior malleolar involvement, ensuring comprehensive preoperative assessment and appropriate fixation strategies are implemented to optimize outcomes.

This study has several limitations. First, its single-center retrospective design results in a patient population, injury severity, and referral patterns that reflect regional characteristics, thereby limiting the generalizability of the findings to other populations or healthcare systems. Second, although a standardized tibial template was used for registration, individual anatomical variations in the distal tibia may introduce subtle discrepancies between mapped fracture lines and actual fracture trajectories. Third, the current fracture line mapping technique only depicts fracture paths on the distal tibial surface and does not capture occult fracture lines within the cancellous bone. Finally, this study does not provide specific recommendations regarding classification, internal fixation methods, or surgical planning for spiral tibial shaft fractures involving the distal articular surface. Future research integrating anatomical and biomechanical insights is required to develop evidence-based, rigorous clinical guidelines.

## Conclusion

Based on CT data from 117 cases of spiral tibial shaft fractures involving the distal articular surface, this study systematically elucidated the incidence of intra-articular extension. For the first time, three-dimensional fracture line mapping combined with heatmap visualization was employed to depict a modular, region-specific distribution pattern across the distal articular surface. The results revealed that the posterior malleolus was the most frequently involved region (72.6%), while the anterior malleolus (39.3%) and medial malleolus (24.9%) also demonstrated non-negligible involvement. Notably, the presence of two linear high-density zones in the anterior region provides intuitive evidence of a previously underestimated fracture risk. Despite limitations inherent to its single-center, retrospective design, this study lays a visual foundation for the development of a unified classification system spanning the tibial shaft and ankle joint. Future multicenter prospective studies incorporating anatomical and biomechanical validation are warranted to translate these heatmap findings into individualized surgical planning, with the ultimate goal of optimizing operative strategies and improving both safety and clinical outcomes.

## Data Availability

The datasets generated and/or analysed during the current study are available from the corresponding author on reasonable request.
